# The Transcriptome Signature of the Receptive Bovine Uterus Determined at Early Gestation

**DOI:** 10.1371/journal.pone.0122874

**Published:** 2015-04-07

**Authors:** Mario Binelli, Saara C. Scolari, Guilherme Pugliesi, Veerle Van Hoeck, Angela M. Gonella-Diaza, Sónia C. S. Andrade, Gustavo R. Gasparin, Luiz L. Coutinho

**Affiliations:** 1 Laboratório de Fisiologia e Endocrinologia Molecular, Department of Animal Reproduction, FMVZ-USP, Pirassununga, Sao Paulo, Brazil; 2 Laboratório de Genética Animal, Departamento de Zootecnia, ESALQ-USP, Pirassununga, Sao Paulo, Brazil; University of Quebec at Trois-Rivieres, CANADA

## Abstract

Pregnancy success is critical to the profitability of cattle operations. However, the molecular events driving the uterine tissue towards embryo receptivity are poorly understood. This study aimed to characterize the uterine transcriptome profiles of pregnant (P) versus non-pregnant (NP) cows during early pregnancy and attempted to define a potential set of marker genes that can be valuable for predicting pregnancy outcome. Therefore, beef cows were synchronized (n=51) and artificially inseminated (n=36) at detected estrus. Six days after AI (D6), jugular blood samples and a biopsy from the uterine horn contralateral to the ovary containing the corpus luteum were collected. Based on pregnancy outcome on D30, samples were retrospectively allocated to the following groups: P (n=6) and NP (n=5). Both groups had similar plasma progesterone concentrations on D6. Uterine biopsies were submitted to RNA-Seq analysis in a Illumina platform. The 272,685,768 million filtered reads were mapped to the *Bos Taurus* reference genome and 14,654 genes were analyzed for differential expression between groups. Transcriptome data showed that 216 genes are differently expressed when comparing NP versus P uterine tissue (*P*adj≤0.1). More specifically, 36 genes were up-regulated in P cows and 180 are up-regulated in NP cows. Functional enrichment and pathway analyses revealed enriched expression of genes associated with extracellular matrix remodeling in the NP cows and nucleotide binding, microsome and vesicular fraction in the P cows. From the 40 top-ranked genes, the transcript levels of nine genes were re-evaluated using qRT-PCR. In conclusion, this study characterized a unique set of genes, expressed in the uterus 6 days after insemination, that indicate a receptive state leading to pregnancy success. Furthermore, expression of such genes can be used as potential markers to efficiently predict pregnancy success.

## Introduction

Subfertility, mainly due to high rates of early embryonic death, is threatening the efficiency and sustainability of the beef cow industry [1, 2]. A non-receptive uterine environment may be a key contributor to the disappointing fertility rates, as the majority of pregnancy losses occur prior to embryo implantation [3]. Therefore, strategies optimizing the reproductive performance of beef cows, aiming to increase rates of embryonic survival, depend on the identification of factors that determine the receptive endometrium.

The uterus becomes receptive for a limited period of time under the influence of steroid hormones and paracrine signals from the developing embryo. At the moment of embryo arrival, the endometrium displays several characteristics that favor the contact and communication, which coincides with days 5–6 of pregnancy [4]. More specifically, the embryo leaves the oviduct and enters the anterior region of the uterus ipsilateral to the ovary containing the corpus luteum, where it will continue its further development supported by the uterine secretions until implantation [4]. As the secretions from the endometrium, i.e. the histotroph, provide the micro-environment in which the conceptus will grow and develop, changes in uterine fluid metabolite levels can be determinant for the fate of the young and developing embryo [5, 6].

Interestingly, up to the time of maternal recognition of pregnancy, progressive changes in the endometrial transcriptome occur independently of the presence of the conceptus [7, 8]. This indicates that receptivity depends on characteristics of the maternal reproductive tract that provide the optimal environment for the early developing embryo. Search continues for key signal determining maternal receptivity towards the embryo. Although several molecules have been put forth as key factors contributing to a receptive uterus, such as adhesion molecules, cytokines and growth factors [9, 10, 11], none of these molecular markers have yet been proven clinically useful in the assessment of functional receptivity.

Currently, a definition of a receptive versus a non-receptive uterine environment remains incomplete. Recent developments in high throughput sequencing technologies provided clues about global trends in gene expression that might offer a prognostic approach to screen and select recipient animals with endometrial characteristics that favor receptivity. Such large scale approaches resulted in highly relevant information at the molecular level determining human uterine receptivity [12–15] which has been compiled to develop an applied prognostic tool for human uterine receptivity assessment, solely based in transcriptome signatures [16, 17].

In cattle, current attempts to describe uterine characteristics favoring maintenance of pregnancy are based on information collected from non-inseminated (and thus NP) cows [18] that makes it difficult to extrapolate and/or link these results to probability of pregnancy. Moreover, several studies that attempted to study the receptivity issue focused on uterine characteristics at time windows that do not coincide with the window of embryonic recognition, such as before [19, 20] or after [21] the moment of embryo reception in the uterus.

Studies screening the endometrial characteristics of the uterus at around the precise time it receives the embryo, i.e., around day 6-post insemination, are lacking. However, such information is highly needed to design strategies in order to tackle the problem of early embryonic mortality in the beef cow industry. Therefore, the present study aimed to perform a whole transcriptome characterization of uterine tissue collected at day 6 post artificial insemination (AI) in beef cattle. A retrospective approach linking pregnancy outcome to gene expression signatures on the day 6 uterine tissues provided a basis for the understanding of the molecular requirements of uterine receptivity.

## Materials and Methods

### Animals

The experiment was carried out in accordance with the Ethics and Animal Handling Committee of the University of São Paulo. It was conducted at the research farm of the University of São Paulo (USP) in Pirassununga, SP, Brazil, using 51 clinically healthy, cycling, multiparous, non- lactating Nellore cows (*Bos indicus*), 4 to 10 years old, with a body condition score between 3 and 4 (scale: 0, emaciated; 5, obese), maintained under the same pasture regimen supplemented with sugar cane and/ or corn silage, concentrate and free access to water and mineralized salt.

### Reproductive management and experimental design

Estruses were synchronized (n = 51) by injecting two im doses of prostaglandin F2α (PGF2α) analogue (500 μg sodium cloprostenol; Sincrocio, Ouro Fino Animal Health, Cravinhos, Brazil) 14 days apart. At the time of the second PGF2α injection, animals were equipped with an Estrotect (Rockway, Inc., USA) device, and animals were observed twice daily until the sixth day after the last PGF2α injection for standing heat. Ultrasound exams were performed with the aid of a duplex B-mode (gray-scale) and Color-Doppler instrument (MyLab30 Vet Gold; Esaote Healthcare, São Paulo, SP, Brazil) equipped with a multi-frequency linear transducer 12 hours after the animals displayed estrus in order to confirm the presence and location of a pre-ovulatory follicle and AI was performed. Six days post-AI, an endometrial biopsy was collected from the uterine horn contralateral to ovulation, as determined by the presence of a corpus luteum (CL). The biopsy procedure was described in detail elsewhere [22]. Once collected, the biopsy was placed in cryotubes and immediately immersed in liquid nitrogen, then transferred to a—80°C for storage. Blood sampling for determination of progesterone concentrations was performed at time of biopsy and processed as described by Mesquita et al. [23]. After the biopsy procedure, cows received two 1 g doses of the anti-inflammatory medication flunixin meglumine (Desflan, Ouro Fino Animal Health, Cravinhos, Brazil) in a 24 hour interval and one dose of a Penicillin/Streptomycin based antibiotic (Penfort Reforçado, Ouro Fino Animal Health, Cravinhos, Brazil). Plasma P4 concentrations were measured by a solid-phase radioimmunoassay (Coat-A-Count; Siemens Medical Solutions Diagnostics, Los Angeles, USA). The animals were observed for signs of abnormal vaginal secretions and/or discomfort after the biopsy. Pregnancy diagnosis was performed on days 22 and 30 post-AI via trans-rectal ultrasonography exam.

### Animal selection for RNA-Seq

RNA-Seq was performed on samples selected retrospectively based on the day 30 pregnancy diagnosis (n = 6 samples from each P and NP animals). Animals were selected based on two criteria: plasma progesterone concentrations on day 6 must had been within a 1.5 ng/mL interval among all animals and, ovulations must had been confirmed within 12 hours after AI as determined by ultrasound exam.

### RNA isolation and cDNA synthesis

Frozen uterine samples (30 mg) were macerated in liquid nitrogen using a stainless steel apparatus, immediately mixed with buffer RLT from the RNeasy Mini columns kit (Qiagen, São Paulo, SP, Brazil) and further DNA, RNA and protein extractions performed using a commercial purification kit (Qiagen AllPrep DNA/RNA/PROTEIN Mini Kit, São Paulo, SP, Brazil) according to the manufacturer’s recommendations. RNA purity and concentration were determined by spectrophotometry (Thermo Fisher Scientific, Suwanee, GA, USA) and RNA integrity was assessed using the 2100 Bioanalyzer (Agilent Technologies Brasil Ltda, São Paulo, SP, Brazil). RNA integrity numbers (RIN) of individual samples ranged from 7 to 8.

The synthesis of deoxyribonucleic acid (cDNA) was performed by reverse transcription using the High Capacity cDNA Reverse Transcription Kit (Life Technologies, Frederick, Maryland, USA) according to manufacturer’s instructions.

#### RNA-Seq and Analysis

For the transcriptome analyses of expression patterns in P and NP animals, cDNA was generated using a routine RNA library preparation TruSeq protocol developed by Illumina Technologies (San Diego, CA) using 1 μg of total RNA as input. Using the kit, mRNA was first isolated from total RNA by performing a polyA selection step, followed by construction of paired-end sequencing libraries with an insert size of about 300 bp. Briefly, polyA selected RNA was cleaved as per Illumina protocol and the cleaved fragments were used to generate first strand cDNA using SuperScript II reverse transcriptase and random hexamers. Subsequently second strand cDNA was synthesized with RNaseH and DNA polymerase enzyme. Adapter ligation and end repair steps followed second strand synthesis. Resulting products were amplified via PCR and cDNA libraries were then purified and validated using the Bioanalyzer 2100 (Agilent Technologies). Paired-end sequencing was performed using the Illumina HiScanSQ platform. Samples were multiplexed with unique six-mer barcodes and run on multiple lanes to obtain 2 x 100 bp reads.

The paired end (PE) reads were filtered using the Seqyclean v1.4.13 package (https://bitbucket.org/izhbannikov/seqyclean) which removed all reads with a mean quality under 26 and removed the primer and vector contaminants using the UniVec database (http://www.ncbi.nlm.nih.gov/tools/vecscreen/univec/). The reads were mapped using the local alignment with Bowtie2 v2.1.0 [24] against the masked *Bos taurus* genome masked assembly (*Bos taurus* UMD3.1) and read counts were obtained using HTSeq-count v0.5.4p2 (http://www-huber.embl.de/users/anders/HTSeq/doc/count.html). From the six biopsies collected in the NP cows and used for RNA-Seq, one sample did not correctly align with the bovine genome and was consequently omitted from the analysis.

The differential expression analysis of the RNA-Seq data was performed with package DESEq2 v1.12.1 [25], from R [26]. The normalized counts were then obtained for each annotated gene. After normalization, the log2 fold change was obtained through a general linear model approach, the significance test was followed by the FDR-Benjamini-Hochberg [27] correction for multiple tests. Genes with the normalized readcounts <5 across all samples were filtered out from the analysis. The enrichment analyses of different functional databases was done using the functional annotation tool of the Database for Annotation, Visualization, and Integrated Discovery (DAVID Bioinformatics Resources 6.7, NIAID/NIH) using as background the genes used on the differential expression analysis [28]. The total list of genes derived from P and NP endometrium was subjected to gene ontology annotation. From the total list, genes involved in different cellular and molecular processes could be identified.

### Quantitative Real-time Polymerase Chain Reaction

Nine of the differentially regulated genes as well as exhibiting the highest fold changes, were selected from the RNA-Seq results for qRT-PCR analysis. Primers for each selected gene were designed using PrimerQuestQM tools (IDT; http://idtdna.com/Scitools/Applications/Primerquest). Amplicon sequence identity was confirmed with NCBI Basic Local Alignment Search Tool software (Blast http://blast.ncbi.nlm.nih.gov/Blast.cgi). Quantitative Real-Time PCR was performed using the StepOnePlus Applied Biosystem Real-Time PCR System and amplification was conducted in triplicate. Reactions were performed in a final volume of 20μL using 10.0 μL of Power SYBR Green PCR Master Mix (Warrington, UK), 10 μM of each primer (forward and reverse), and 4 μL of cDNA (diluted 1:40). Temperature profiles comprised an initial denaturation step at 95°C for 10 min, and 40 cycles with denaturation at 95°C for 15 sec and annealing at 60°C for 1 min. Cyclophilin was used as the normalizing reference gene. Relative expression levels of the selected target genes were calculated with the LinRegPCR software method. The cycle threshold (Ct) values determined for the target genes were normalized against the reference gene. PCR product identity was confirmed by sequencing. Primers designed for these genes are in Table 1.

**Table 1:** List of primers used to validate RNA-Seq results.

Gene	Forward	Reverse
*ADAM12*	CTCCATAAGTCAGGACCCATTC	CGGTGGTTCCTTGGAAATAAAC
*ICAM1*	AGACCCTGAAGTGCGAGGCT	TATTCTGGCCGTGGAGCACGTT
*FRAS1*	AGGAACTACTGGGAGATCAG	GGCTGAGCTGGAAGAATAAA
*DIO2*	CATCGTGCAGAGACAGAAA	AACCAGCTAACCAGCTAATC
*PNMT*	ATGTCAAGGGCATCTTCTTC	CTCAGACAACAGGGAGTCTA
*PTGES*	GCTGCGGAAGAAGGCTTTTGCC	GGGCTCTGAGGCAGCGTTCC
*CYP2U1*	TGTCACTGCACCCCAACATT	CCCGCTTCCCTGTTTTCTCT
*RIMKLA*	CAGCTCTGGTTCCTGACAGA	GAAAGTGGTGAGCGCCTTCT
*SERPINA11*	GGACCTTGCTAGGATGGGG	TGATTTTCTGGTAGGCGGGG

Statistical analyses of qRT-PCR data were performed using Student's paired t-test.

## Results

### Animals

A total of 51 cows were synchronized and 36 displayed estrus, ovulated and hence, received AI. Progesterone concentrations on day 6 post-AI varied between 2.2 and 9.9 ng/ml, and cows within the 4.3 to 5.8 ng/mL range were selected for RNA sequencing P (n = 6) and NP (n = 5).

### RNA-Seq analyses

**Fig 1:**
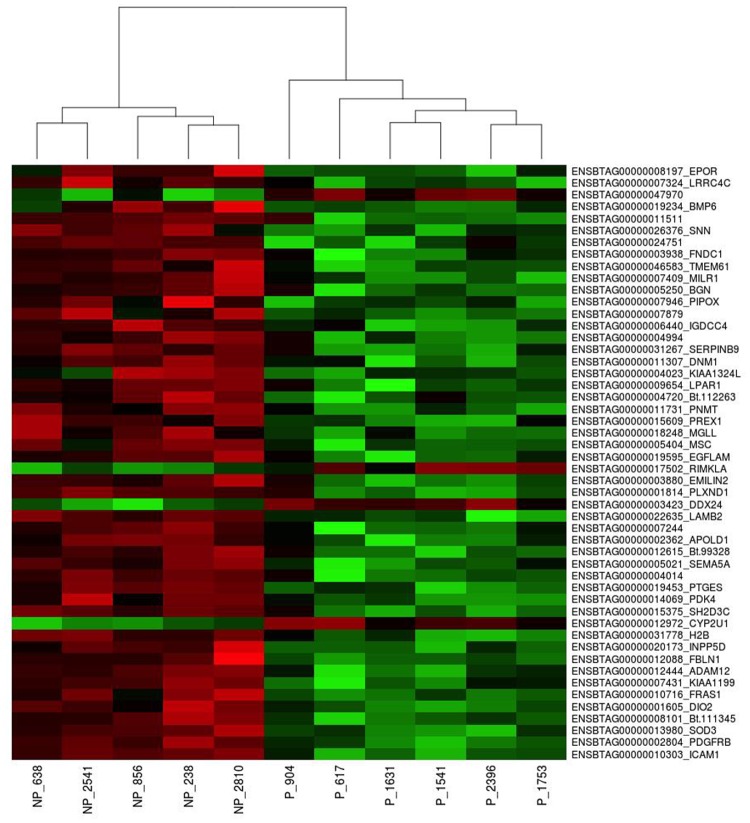
Pregnant and non-pregnant uterine differential gene expression. Heatmap and hierarchical clustering of all 11 samples based on the most differentially expressed genes (Padjusted).

RNA-Seq analysis was performed in six cows per group, but one cow in the NP group was omitted due to sequencing issues. It resulted in 334,065,816 millions of total paired-end reads and 272,685,768 million filtered reads. The reads were mapped to the *Bos taurus* UMD3.1 genome and, following the filtering, 14,654 genes were effectively analyzed for differential expression between groups. All reads sequences were deposited in the Sequence Read Archive (SRA) of the NCBI (http://www.ncbi.nlm.nih.gov/sra/; accession numbers in S1 Dataset.). An overview of the gene expression data has been deposited in NCBI’s Gene Expression Omnibus (GEO) and is accessible through GEO Series accession number GSE65117 (S1 Materials.). Transcriptome data showed that 216 genes are differently expressed when comparing NP versus P uterine tissue (*P*adj<0.1). More specifically, 36 genes showed a significantly up-regulated expression for P cows and 180 were up-regulated for NP cows. In Tables 2 and 3, a list is provided with the top-annotated 20 genes (based on Log2Fold-Change) with up-regulated expression in uterine biopsies from P cows and NP cows. All differentially expressed genes are presented on S2 Dataset.). Fig 1 shows the global gene expression profiles among all samples using a heatmap along with an unsupervised linkage hierarchical clustering using the 50 differentially expressed genes (with the lowest p adjusted values) segregating endometrial samples from both groups.

**Table 2:** List displaying the 20 top-annotated genes (based on Log2Fold-Change) with up-regulated expression in uterine biopsies from non-pregnant cows *Padj*≤0.1.

	Gene Name	Symbol	*Padj*	Log2Fold-Change
1	**Fraser syndrome 1**	*FRAS1*	0.0001	1.7644
2	**Deiodinase, Iodothyronine, type II**	*DIO2*	0.0001	1.7344
3	**ADAM metallopeptidase domain 12**	*ADAM12*	0.0004	1.5344
4	**KIAA1324-like ortholog**	*KIAA1324L*	0.0096	1.4320
5	**Leucine Rich Repeat Containing 4C**	*LRRC4C*	0.0140	1.4019
6	**Transmembrane Protein 61**	*TMEM61*	0.0115	1.3626
7	**Adenylate Kinase 5**	*AK5*	0.0266	1.3616
8	**Pipecolic Acid Oxidase**	*PIPOX*	0.0108	1.3346
9	**KIAA1199 Ortholog**	*KIAA1199*	0.0004	1.3307
10	**UL16 Binding Protein 3**	*ULBP3*	0.0357	1.3268
11	**apolipoprotein L domain containing 1**	*APOLD1*	0.0019	1.2902
12	**phenylethanolamine N-methyltransferase**	*PNMT*	0.0071	1.2735
13	**Gprotein-coupled receptor 65**	*GPR65*	0.0383	1.2555
14	**NK6 Homeobox 1**	*NKX6-1*	0.0554	1.2445
15	**Odd-skipped related 1**	*OSR1*	0.0242	1.2203
16	**Mast Cell immunoglobulin-like receptor 1**	*MILR1*	0.0109	1.2038
17	**Musculin**	*MSC*	0.0060	1.2024
18	**Collagen, Type VII, Alpha 1**	*COL7A1*	0.0637	1.1958
19	**Solute Carrier Family 30**	*SLC30A2*	0.0800	1.1879
20	**Synaptotagmin I**	*SYT1*	0.0809	1.1790

**Table 3:** List displaying the 20 top-annotated genes (based on Log2Fold-Change) with up-regulated expression in uterine biopsies from pregnant cows *Padj* ≤0.1.

	Gene Name	GeneSymbol	*Padj*	Log2Fold-Change
1	**Ribosomal Modification protein rimK-like family member A**	*RIMKLA*	0.0048	-1.3978
2	**serpin peptidase inhibitor, clade A member 11**	*SERPINA11*	0.0142	-1.3242
3	**mannan-binding lectin serine peptidase 1**	*MASP1*	0.0500	-1.2715
4	**DEAD (Asp-Glu-Ala-Asp) box polypeptide 25**	*DDX25*	0.0742	-1.2070
5	**UDP glucuronosyltransferase 2 family, polypeptide A3**	*UGT2A3*	0.0583	-1.1981
6	**TRAPPIN-4**	*BTRAPPIN-4*	0.0983	-1.1394
7	**Regulator of G-protein Signaling 17**	*RGS17*	0.0926	-1.1355
8	**transmembrane protein 213**	*TMEM213*	0.0926	-1.1294
9	**mesothelin**	*MSLN*	0.0809	-1.0832
10	**cytochrome P450, family 2, subfamily A, polypeptide 13-like**	*CYP2A13*	0.1077	-0.9746
11	**growth factor receptor-bound protein 14**	*GRB14*	0.0487	-0.9473
12	**X-linked Kx blood group**	*XK*	0.0961	-0.7959
13	**cellular retinoic acid binding protein 1**	*CRABP1*	0.0550	-0.7955
14	**ring finger protein 219**	*RNF219*	*0*.*1031*	-0.6704
15	**7-dehydrocholesterol reductase**	*DHCR7*	0.1031	-0.5550
16	**alkB, alkylation repair homolog 3**	*ALKBH3*	0.0877	-0.5452
17	**cytochrome P450, family 2, subfamily U, polypeptide 1**	*CYP2U1*	0.0007	-0.5133
18	**ATPase, Na+/K+ transporting, alpha 1 polypeptide**	*ATP1A1*	0.0428	-0.5038
19	**ubiquitin-conjugating enzyme E2N**	*UBE2N*	0.0134	-0.4785
20	**dynein, light chain, Tctex-type 1**	*DYNLT1*	0.0418	-0.4779

### Functional enrichment analysis and pathway analysis

**Fig 2:**
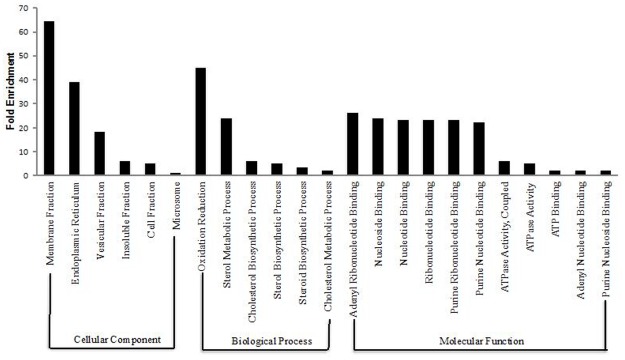
GO analysis and enrichment. Enriched categories for cellular component, molecular function, and biological process for the pregnant group.

**Fig 3:**
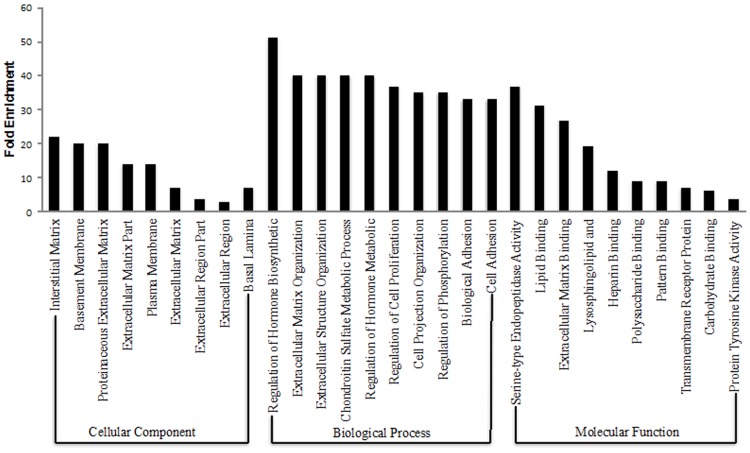
GO analysis and enrichment. Enriched categories for cellular component, molecular function, and biological process for the non-pregnant group.

In order to gain more insight into key processes that may possibly explain functional differences between the P and NP uteri, functional annotation analyses were carried out on enriched genes identified in each group using DAVID Bioinformatics Resources 6.7 [http://david.abcc.ncifcrf.gov/home.jsp]. Functional annotation clustering revealed 5 clusters in the P group with highest enrichment score of 1.53 and pathways such as microsome, vesicular, membrane, insoluble and cell fraction (*CYP2U1*, *Grb14*, *ATP1A1*). ATP binding (*DDX25*, *ATP1A1*, *UBE2N*) and steroid biosynthetic process (*DHCR7*, *MVD*) were significantly enriched (Fig 2). The analysis revealed that the overall genomic expression profiles was substantially increased for the Gene Ontology (GO) terms and KEGG pathways (*P*<0.05 after Benjamini correction) involved in a wide range of biological processes, cellular component and molecular function for the NP in comparison to the P group as shown (Fig 3). Functional annotation clustering for the NP group revealed 31 clusters with highest enrichment score of 7.2. The most significantly enriched pathways were responsible for endometrial morphological changes and pointed especially to pathways involved in the extracellular matrix region, matrix and remodeling. Genes included *ADAM12*, *ADAMTS1*, *TIMP3*, *BGN*, *EGFLAM*, *Angpt4*, *AGRN and BMP4*.

### qRT-PCR Validation

The transcript levels of nine genes were re-evaluated using quantitative real-time PCR (qRT-PCR). RNA from the same animals assigned to the RNA-Seq analysis (P, n = 6; NP, n = 6) were used for the qRT-PCR validation experiment. Moreover, transcript levels from uterine biopsies of 15 additional animals (P, n = 5; NP, n = 10), of different biological origin but from the same experiment as the samples evaluated with the RNA-Seq procedures, were quantified. Overall, there was an agreement for the results obtained from RNA-Seq and qRT-PCR (Table 4).

**Table 4:** Validation of RNA-Seq gene expression data by qRT-PCR.

Gene Symbol	RNA-Seq (n = 11^a^)			qPCR (n = 11^a^)			qPCR (n = 15^b^)		
	Fold Change	log2 Fold Change	*P* value	Fold Change	log2 Fold Change	*P* value	Fold Change	log2 Fold Change	*P* value
***FRAS1***	3.40	1.76	0.0001	2.04	1.02	0.056	2.92	1.54	0.015
***DIO2***	3.33	1.73	0.0001	3.51	1.81	0.0002	3.64	1.86	0.009
***ADAM12***	2.90	1.53	0.0004	3.2	1.67	0.004	2.12	1.08	0.001
***PNMT***	2.4	1.27	0.0071	2.38	1.25	0.01	3.2	1.67	0.05
***ICAM1***	2.15	1.10	0.00000	2.6	1.37	0.0002	2	1	0.004
***PTGES***	2.13	1.09	0.0008	2.09	1.06	0.03	2.6	1.37	0.01
***RIMKLA***	0.38	-1.39	0.0048	0.38	-1.40	0.004	0.73	-0.45	0.002
***SERPINA11***	0.40	-1.32	0.0142	0.29	-1.79	0.0008	0.25	-2	0.002
***CYP2U1***	0.70	-0.51	0.0007	0.31	-1.69	0.001	0.35	-1.51	0.002

^a^ uterine biopsies of the same origin.

^b^ indicates biologically different uterine biopsies.

Fold changes, log2 Fold Changes and *p* values of gene expression in non-pregnant versus pregnant uterine biopsies are presented.

## Discussion

The endometrium plays a central role among the reproductive tissues in the context of early embryo-maternal communication and a successful pregnancy depends on a complex series of endometrial molecular and cellular events. The factors responsible for the initial interaction between maternal and embryonic tissues, leading to the establishment of pregnancy, remain poorly understood. Published studies that thrived or attempted to define uterine markers for pregnancy, selected the markers based on endometrial tissue collected during a NP cycle, in order to not interfere with ongoing pregnancy [18, 10]. In the present study, we thrived to provide, for the first time, information on the day 6 endometrial transcriptome signature from uterine biopsies that were collected in the same cycle of the AI. This means that, data on pregnancy outcome were retrospectively linked with endometrial characteristics. Samples were collected from contralateral uterine horn in relation to the pregnancy without a negative effect on pregnancy outcome as previously reported [22].

Using RNA sequencing methods, 14.654 reference genes were effectively analyzed for differential expression between P and NP uterine tissue. Transcriptome data revealed that 216 genes were differently expressed when comparing uterine tissue from P and NP cows. Interestingly, only 36 of the differently expressed genes displayed an up-regulated expression in the P uterine tissue, whereas up to 180 genes displayed a down-regulated expression. This might indicate that a favorable uterine environment for maintenance of pregnancy might require a “quiet” state. This can be highly relevant to consider as previous work from Baumann [29] reported that pre-implantation embryo survival is best served by a relatively low level of metabolism; a situation achieved by reducing the concentrations of nutrients in its environment and encouraging the use endogenous resources. Interestingly, Salilew-Wondim et al. (2010) previously investigated whether pre-transfer endometrial and embryo gene expression patterns have a direct relation with upcoming pregnancy success. A global endometrial transcriptome analysis was performed using endometrial and embryo biopsy technology; endometrial samples were collected from Simmental heifers at day 7 and 14 of the estrous cycle prior to embryo transfer. The results revealed that at day 7 of the estrous cycle, the endometrial gene expression pattern of heifers whose pregnancy resulted in calf delivery was significantly different than those resulting in no pregnancy. The fact that our RNAseq results point to similar findings, but now in endometrial biopsies of the same cycle of the pregnancy, highlights the potential of screening endometrial gene expression profiles around the moment of embryo arrival in the uterus as tool to predict pregnancy chances.

However, bioanalyses of the transcriptome data reveals that main pathways being over-expressed in pregnancy are related to intracellular trafficking and steroid biosynthetic activities. Genes encoding enzymes involved in steroid biosynthesis, such as *CYP450* and *DHCR7*, belong to the top-20 list with up-regulated genes in the uterus of P cows. Therefore, one could assume that such up-regulated expression of lipogenesis genes, which might result in increased endometrial anabolic activities, are needed contributions to the histotroph composition towards the young and developing pre-implantation embryo [19]. The latter anabolic tasks requires a certain level of differentiation at the molecular level, which might be corroborated by the up-regulated expression of the *SERPINA11*, which is included in the top-20 list of up-regulated genes in the P cows. Serpins exert supportive functions towards the achievement of a well-prepared uterine environment for passing gametes, especially sperm [30].

The pathway regulating cell proliferation is one of the main down-regulated pathways in the day 6 endometrial tissue favoring pregnancy. The top-20 list, compiling the genes with down-regulated expression in the P uterus, contains several genes involved in cell proliferation functions such as *KIAA1324L* and *KIAA1199*. In line with this, [31] recently suggested that a receptive endometrium should display characteristics of a tissue that surpassed the proliferative status, in order to provide a more mature, differentiated micro-environment towards the embryo. Proliferating activities could jeopardize the crucial first maternal recognition events in the uterus and thereby disrupt the maternal-embryonic cross-talk, a prerequisite to safeguard subsequent pregnancy as been shown in murine and human studies [32].

Other important pathways in day 6 P versus NP uterine tissue were related to extracellular matrix (ECM) remodeling and cell adhesion processes. Indeed, *COL7A1* and *ADAM12*, which are genes associated with extracellular matrix formation, belong to the top-20 list of genes with down-regulated expression in the P uterus. Previous murine, human and ovine reports [33–35] already documented on the importance of the latter pathway: maternal-embryonic adhesion involves a complex sequence of signaling events, consisting in the acquisition of adhesion ligands together with the loss of inhibitory components, which are crucial to the establishment of pregnancy. It is likely that each of these, when appropriately expressed or inhibited, contributes to endometrial receptivity or non-receptivity to an implanting conceptus [36, 37]. Excessive amounts of ECM could possibly inhibit the initial embryonic contact with maternal endometrial tissue [38].

Several research groups helped to enlighten the endometrial characteristics that are favourable for pregnancy success [5, 20, 21, 39, 40]. Most available information, so far, is based on tissue collected at timings beyond the first maternal recognition events in cattle, such as day 16 [21] and day 17 of pregnancy [40]. This serves to understand the molecular basis triggering the process of implantation, though does not allow resulting information to be used as screening tool to select recipient cows.

Being primarily based on transcriptome information, the data described in the present study should be interpreted with caution. It is not clear whether all transcripts will be translated and/or even modified due to post-translational influences [41]. The importance of this consideration has been recently emphasized by Walker *et al*. [42]: DNA methylation is involved in early pregnancy events, which might point towards potential post-transcriptional alterations. However, the use of transcriptome platforms allows screening for potential markers that are determinant for the maternal receptive signature. Using a clustering approach in the present study, several specific candidate genes have been evaluated. Candidate genes were selected according to their ranking from the most significant differentially expressed genes. Although the genes of interest had not been previously described in relation to the bovine endometrium in earlier studies, their high levels of expression might be crucial for achieving a uterine receptive state. This highlights the need for additional research to implement this set of receptivity marker transcripts into a hands-on clinical tool that should select the optimal recipient cows for embryo transfer.

Finding markers for uterine receptivity is of cross-species interest. Despite increasing global experience with advanced reproductive technologies, the majority of human *in vitro* fertilization attempts remain unsuccessful, most likely on the basis of implantation failure [12, 43]. Pregnancy rates after human embryo transfer remain low and several researchers tempted to design tools to evaluate human receptivity markers [44].

In conclusion, this study offers a valuable basis for understanding genes with potential functional and clinical relevance in endometrial receptivity and results in a unique set of transcripts, expressed in the bovine uterus as early as 6 days after AI. The abundance of these gene transcripts can be indicative for a receptive state leading to pregnancy success. It is proposed that as regulation of these markers is elucidated, their expression may be manipulated to improve endometrial receptivity and fertility.

## Supporting Information

S1 Dataset Lists displaying all genes with up-regulated expression in uterine biopsies from non-pregnant cows (Table A.) and from pregnant cows (Table B.) (*Padj*<0.1). (DOCX)

S1 Materials Sequence Read Archive (SRA) accession numbers of the filtered reads resulting from the RNAseq. (DOCX)
