# Supplementary material for: The Transcriptome Signature of the Receptive Bovine Uterus Determined at Early Gestation
Source: PLoS One. 2015 Apr 7;10(4):e0122874. doi: 10.1371/journal.pone.0122874 (PMC4388694; doi:10.1371/journal.pone.0122874)
Supplement: S1 Dataset — (DOCX) [file pone.0122874.s001.docx]

**S1 Dataset.** Lists displaying all genes with up-regulated expression in uterine biopsies from non-pregnant cows (Supporting Table 2A) and from pregnant cows (Supporting Table 2B) (Padj<0.1).

**Supporting Table A.** List displaying all genes with up-regulated expression in uterine biopsies from non-pregnant cows (*Padj*<0.1). **id: Ensembl number identification; lfcSE: log2foldchange standard error; padj: p-value adjusted after Benjamini-Hochberg correction for multiple tests.**

| id | Gene.Symbol | baseMean | log2FoldChange | lfcSEpadj |
| --- | --- | --- | --- | --- |
| ENSBTAG00000010303 | ICAM1 | 239,6435 | 1,1064 | 0,15760,0000 |
| ENSBTAG00000002804 | PDGFRB | 2037,7445 | 0,7877 | 0,11960,0000 |
| ENSBTAG00000013980 | SOD3 | 211,8684 | 0,9439 | 0,15620,0000 |
| ENSBTAG00000008101 | Bt.111345 | 153,1394 | 1,1920 | 0,20370,0000 |
| ENSBTAG00000001605 | DIO2 | 24,8384 | 1,7344 | 0,31010,0001 |
| ENSBTAG00000010716 | FRAS1 | 17,0973 | 1,7644 | 0,31530,0001 |
| ENSBTAG00000007431 | KIAA1199 | 237,3309 | 1,3307 | 0,25500,0004 |
| ENSBTAG00000012444 | ADAM12 | 419,9302 | 1,5344 | 0,29470,0004 |
| ENSBTAG00000012088 | FBLN1 | 5161,1970 | 0,6615 | 0,12950,0005 |
| ENSBTAG00000020173 | INPP5D | 90,0044 | 0,9083 | 0,18020,0006 |
| ENSBTAG00000031778 | H2B; HIST1H2BL; LOC781410; HIST1H2BD | 83,2586 | 1,1520 | 0,22870,0006 |
| ENSBTAG00000015375 | SH2D3C | 207,7655 | 0,7199 | 0,14460,0007 |
| ENSBTAG00000019453 | PTGES | 27,4011 | 1,0929 | 0,22110,0008 |
| ENSBTAG00000014069 | PDK4 | 572,5771 | 1,1776 | 0,23830,0008 |
| ENSBTAG00000005021 | SEMA5A | 1381,2089 | 0,8993 | 0,18440,0010 |
| ENSBTAG00000004014 | NA | 1585,8126 | 0,9609 | 0,19720,0010 |
| ENSBTAG00000012615 | Bt.99328 | 580,6566 | 0,9463 | 0,19780,0014 |
| ENSBTAG00000002362 | APOLD1 | 368,5749 | 1,2902 | 0,27390,0019 |
| ENSBTAG00000007244 | NA | 1368,5778 | 1,1613 | 0,25410,0036 |
| ENSBTAG00000022635 | LAMB2 | 2027,3089 | 0,4154 | 0,09120,0037 |
| ENSBTAG00000001814 | PLXND1 | 1080,4926 | 0,7064 | 0,15670,0041 |
| ENSBTAG00000003880 | EMILIN2 | 2339,9406 | 1,0754 | 0,23880,0041 |
| ENSBTAG00000019595 | EGFLAM | 447,2406 | 1,0909 | 0,24500,0048 |
| ENSBTAG00000005404 | MSC | 26,3538 | 1,2024 | 0,27360,0060 |
| ENSBTAG00000018248 | MGLL | 188,2765 | 0,7621 | 0,17390,0061 |
| ENSBTAG00000015609 | PREX1 | 227,4856 | 0,7838 | 0,18040,0071 |
| ENSBTAG00000011731 | PNMT | 30,9461 | 1,2735 | 0,29380,0071 |
| ENSBTAG00000004720 | Bt.112263 | 1302,0932 | 1,0412 | 0,24210,0080 |
| ENSBTAG00000009654 | LPAR1 | 489,6577 | 1,0346 | 0,24150,0084 |
| ENSBTAG00000004023 | KIAA1324L | 123,9528 | 1,4320 | 0,33710,0096 |
| ENSBTAG00000011307 | DNM1 | 374,7305 | 0,8159 | 0,19270,0099 |
| ENSBTAG00000031267 | SERPINB9 | 79,6772 | 0,8771 | 0,20800,0104 |
| ENSBTAG00000007879 | NA | 43,9279 | 0,8678 | 0,20710,0108 |
| ENSBTAG00000004994 | NA | 125,6444 | 0,9514 | 0,22720,0108 |
| ENSBTAG00000006440 | IGDCC4 | 155,4703 | 1,0857 | 0,25880,0108 |
| ENSBTAG00000007946 | PIPOX | 30,9991 | 1,3346 | 0,31900,0108 |
| ENSBTAG00000005250 | BGN | 1541,3817 | 0,9489 | 0,22710,0108 |
| ENSBTAG00000007409 | MILR1 | 18,1035 | 1,2038 | 0,28870,0109 |
| ENSBTAG00000046583 | TMEM61 | 11,2870 | 1,3626 | 0,32810,0115 |
| ENSBTAG00000003938 | FNDC1; LOC783891; LOC100847777 | 2382,0284 | 1,3358 | 0,32360,0125 |
| ENSBTAG00000026376 | SNN | 106,0515 | 0,6208 | 0,15070,0125 |
| ENSBTAG00000024751 | NA | 5,4064 | 1,4472 | 0,35150,0125 |
| ENSBTAG00000011511 | NA | 279,8520 | 0,9256 | 0,22520,0126 |
| ENSBTAG00000019234 | BMP6 | 64,4602 | 1,0821 | 0,26480,0134 |
| ENSBTAG00000014828 | CACNA1A | 122,8761 | 0,8882 | 0,21870,0140 |
| ENSBTAG00000008197 | EPOR | 38,9249 | 0,9616 | 0,23710,0140 |
| ENSBTAG00000020542 | SHE | 95,5340 | 1,0401 | 0,25650,0140 |
| ENSBTAG00000009998 | GALNTL1 | 650,6791 | 1,0799 | 0,26650,0140 |
| ENSBTAG00000016279 | LOC788927 | 119,1077 | 1,1069 | 0,27340,0140 |
| ENSBTAG00000007324 | LRRC4C | 25,4709 | 1,4019 | 0,34620,0140 |
| ENSBTAG00000012405 | PEAR1 | 661,3908 | 0,9516 | 0,23730,0159 |
| ENSBTAG00000015834 | ARHGEF4 | 136,7345 | 0,9026 | 0,22590,0162 |
| ENSBTAG00000013066 | IGF2 | 6118,9648 | 1,0480 | 0,26240,0162 |
| ENSBTAG00000012526 | APBB1IP | 133,5418 | 1,1203 | 0,28040,0162 |
| ENSBTAG00000000917 | Bt.48679 | 686,6299 | 0,5210 | 0,13070,0162 |
| ENSBTAG00000013744 | NA | 204,1072 | 0,6372 | 0,15990,0162 |
| ENSBTAG00000044073 | LOC520070 | 698,6506 | 0,7314 | 0,18400,0166 |
| ENSBTAG00000008250 | SPRY4 | 68,4020 | 0,7069 | 0,17850,0170 |
| ENSBTAG00000003832 | MFAP2 | 885,3411 | 0,8696 | 0,21960,0170 |
| ENSBTAG00000033529 | SCN2B | 49,9240 | 0,9342 | 0,23600,0170 |
| ENSBTAG00000016644 | AQPEP | 39,0462 | 1,1322 | 0,28670,0174 |
| ENSBTAG00000005990 | S1PR1 | 1304,6595 | 0,8387 | 0,21430,0196 |
| ENSBTAG00000015562 | PLBD1 | 574,6424 | 0,9669 | 0,24920,0221 |
| ENSBTAG00000037778 | CXCL3 | 158,1886 | 1,1433 | 0,29510,0224 |
| ENSBTAG00000010123 | APOE | 3672,0131 | 1,0403 | 0,26900,0227 |
| ENSBTAG00000008113 | OSR1 | 1028,0434 | 1,2203 | 0,31710,0242 |
| ENSBTAG00000003793 | NGLY1 | 642,5257 | 0,4168 | 0,10850,0243 |
| ENSBTAG00000013191 | AGRN | 1546,2996 | 0,5162 | 0,13480,0243 |
| ENSBTAG00000008346 | Bt.111029 | 317,5406 | 0,5779 | 0,15080,0243 |
| ENSBTAG00000040128 | FZD4 | 213,9724 | 0,9462 | 0,24670,0243 |
| ENSBTAG00000002680 | TMEM132E | 24,8763 | 1,0460 | 0,27250,0243 |
| ENSBTAG00000011266 | ZBTB16 | 159,3128 | 0,8860 | 0,23150,0244 |
| ENSBTAG00000019347 | PLXDC1 | 1585,7695 | 1,1295 | 0,29560,0244 |
| ENSBTAG00000046595 | NA | 6,3635 | 1,3538 | 0,35430,0244 |
| ENSBTAG00000009851 | ROBO1 | 510,7912 | 0,6440 | 0,16940,0254 |
| ENSBTAG00000017490 | PLD1 | 178,8001 | 0,8313 | 0,21830,0254 |
| ENSBTAG00000011573 | NA | 553,4784 | 1,1251 | 0,29580,0254 |
| ENSBTAG00000017411 | AK5 | 157,2275 | 1,3616 | 0,35960,0266 |
| ENSBTAG00000012247 | MXRA8 | 1271,7185 | 0,6740 | 0,17850,0275 |
| ENSBTAG00000019832 | TGFBR2 | 4271,1696 | 0,7196 | 0,19080,0277 |
| ENSBTAG00000014484 | TMEM26 | 76,4104 | 1,0863 | 0,28920,0290 |
| ENSBTAG00000013919 | BOLA-DRB3 | 984,9608 | 0,7211 | 0,19250,0295 |
| ENSBTAG00000002586 | TCF12 | 4473,1181 | 0,7263 | 0,19400,0295 |
| ENSBTAG00000007665 | NPR3 | 120,8588 | 1,1096 | 0,29630,0295 |
| ENSBTAG00000019343 | MDFI | 57,5662 | 1,1227 | 0,30290,0335 |
| ENSBTAG00000004388 | SLITRK6 | 27,1740 | 1,1406 | 0,30770,0335 |
| ENSBTAG00000010551 | ATP1A2 | 355,1540 | 0,7391 | 0,20040,0350 |
| ENSBTAG00000022150 | MXRA5 | 893,6174 | 0,8681 | 0,23540,0350 |
| ENSBTAG00000037399 | NA | 713,7271 | 0,9070 | 0,24590,0350 |
| ENSBTAG00000026437 | ULBP3 | 18,0530 | 1,3268 | 0,36060,0357 |
| ENSBTAG00000020854 | BCL6B | 159,4524 | 0,8872 | 0,24160,0364 |
| ENSBTAG00000009150 | SPON1 | 7059,3165 | 1,0143 | 0,27740,0382 |
| ENSBTAG00000020589 | ENTPD1 | 350,5697 | 0,8496 | 0,23270,0383 |
| ENSBTAG00000012737 | GPR65 | 11,4508 | 1,2555 | 0,34390,0383 |
| ENSBTAG00000020143 | RDH10 | 772,4397 | 1,1201 | 0,30710,0384 |
| ENSBTAG00000031849 | TMEM119 | 807,5324 | 0,8297 | 0,22830,0393 |
| ENSBTAG00000012514 | PODN | 627,9451 | 0,8456 | 0,23260,0393 |
| ENSBTAG00000021811 | ANGPT4 | 449,3812 | 1,0961 | 0,30150,0393 |
| ENSBTAG00000009331 | CPAMD8 | 208,3146 | 1,0059 | 0,27740,0401 |
| ENSBTAG00000004885 | DDR2 | 480,7240 | 0,5349 | 0,14800,0415 |
| ENSBTAG00000027625 | SFRP1 | 6120,0925 | 1,0693 | 0,29610,0416 |
| ENSBTAG00000004115 | MYLIP | 1433,4471 | 0,7536 | 0,20920,0418 |
| ENSBTAG00000019251 | EPB41L3 | 499,5252 | 0,7680 | 0,21310,0418 |
| ENSBTAG00000006675 | PCSK6 | 741,7253 | 0,9725 | 0,26980,0418 |
| ENSBTAG00000011458 | CPXM1 | 1982,8963 | 1,0009 | 0,28010,0458 |
| ENSBTAG00000003212 | NNAT | 1196,5667 | 1,1029 | 0,30910,0462 |
| ENSBTAG00000047200 | HYAL1 | 121,6201 | 0,4849 | 0,13600,0464 |
| ENSBTAG00000022161 | TLR7 | 19,3885 | 1,0097 | 0,28400,0478 |
| ENSBTAG00000007187 | INF2 | 214,0409 | 0,6988 | 0,19720,0487 |
| ENSBTAG00000000306 | ITK | 32,8183 | 0,7802 | 0,22010,0487 |
| ENSBTAG00000016506 | ST3GAL4 | 773,9090 | 0,4564 | 0,12920,0500 |
| ENSBTAG00000030180 | SHANK3 | 451,4203 | 0,5157 | 0,14620,0500 |
| ENSBTAG00000020939 | PLAC9 | 192,8939 | 0,7330 | 0,20740,0500 |
| ENSBTAG00000008814 | NA | 781,5496 | 0,8487 | 0,24080,0500 |
| ENSBTAG00000030259 | RASGRF2 | 126,9136 | 0,8348 | 0,23800,0525 |
| ENSBTAG00000001176 | LRRN1 | 811,0449 | 1,0273 | 0,29280,0525 |
| ENSBTAG00000007589 | SMAD9 | 71,0029 | 0,8822 | 0,25170,0527 |
| ENSBTAG00000046503 | NA | 66,5024 | 0,5339 | 0,15350,0554 |
| ENSBTAG00000005145 | LOC786652 | 256,4255 | 0,5670 | 0,16290,0554 |
| ENSBTAG00000000494 | PDE4D | 281,7592 | 0,7110 | 0,20460,0554 |
| ENSBTAG00000003809 | PLCD4 | 125,9924 | 0,8268 | 0,23790,0554 |
| ENSBTAG00000044070 | SNX30 | 30,1090 | 0,9950 | 0,28620,0554 |
| ENSBTAG00000005525 | LHX6 | 18,1579 | 1,1475 | 0,32990,0554 |
| ENSBTAG00000002518 | NKX6-1 | 5,0278 | 1,2445 | 0,35800,0554 |
| ENSBTAG00000021444 | TWIST2 | 210,7460 | 1,0910 | 0,31460,0565 |
| ENSBTAG00000012297 | FAM65C | 96,5778 | 1,0289 | 0,29790,0583 |
| ENSBTAG00000013843 | ACVRL1 | 1113,4977 | 0,7615 | 0,22070,0588 |
| ENSBTAG00000045879 | NA | 39,3402 | 0,8275 | 0,24170,0628 |
| ENSBTAG00000030587 | LASP1; LOC787753 | 2571,2428 | 0,3461 | 0,10130,0637 |
| ENSBTAG00000019081 | COL7A1 | 1117,0562 | 1,1958 | 0,35010,0637 |
| ENSBTAG00000019164 | RHOBTB1 | 607,5850 | 0,6344 | 0,18640,0655 |
| ENSBTAG00000011483 | SCARF1 | 273,6111 | 0,7424 | 0,21840,0661 |
| ENSBTAG00000017901 | LAYN | 93,2671 | 0,7633 | 0,22530,0680 |
| ENSBTAG00000013863 | DUSP1 | 1206,1510 | 0,6761 | 0,19990,0688 |
| ENSBTAG00000005799 | LOC781667 | 119,2310 | 0,7073 | 0,20940,0697 |
| ENSBTAG00000010793 | CCDC80 | 5639,2540 | 0,8362 | 0,24780,0701 |
| ENSBTAG00000015304 | ANXA9 | 46,8908 | 0,8387 | 0,24950,0724 |
| ENSBTAG00000009806 | INVS | 624,5543 | 0,3574 | 0,10650,0737 |
| ENSBTAG00000020880 | C10H14orf37 | 266,9797 | 0,8122 | 0,24230,0741 |
| ENSBTAG00000032531 | MUSTN1 | 313,1919 | 0,6261 | 0,18730,0742 |
| ENSBTAG00000034435 | NKD2 | 145,8033 | 0,6628 | 0,19810,0742 |
| ENSBTAG00000001414 | KCTD12 | 82,5403 | 0,6878 | 0,20590,0742 |
| ENSBTAG00000020415 | KLK4 | 99,0956 | 0,9886 | 0,29570,0742 |
| ENSBTAG00000023600 | APOD | 2750,6597 | 0,9723 | 0,29130,0744 |
| ENSBTAG00000014947 | PTPN13 | 434,5654 | 0,5709 | 0,17180,0773 |
| ENSBTAG00000000706 | ADAMTS1 | 1944,2107 | 0,7288 | 0,21970,0774 |
| ENSBTAG00000010927 | CBFA2T3 | 56,2553 | 0,8648 | 0,26050,0774 |
| ENSBTAG00000021015 | FAM196A | 33,9099 | 1,1504 | 0,34680,0774 |
| ENSBTAG00000016885 | LRRTM3 | 12,8233 | 1,1725 | 0,35410,0787 |
| ENSBTAG00000027569 | APBB2 | 558,2642 | 0,5499 | 0,16680,0799 |
| ENSBTAG00000001009 | HCLS1 | 52,2056 | 0,6463 | 0,19600,0799 |
| ENSBTAG00000020459 | TIMELESS | 59,3627 | 0,9425 | 0,28560,0799 |
| ENSBTAG00000018300 | TNFRSF19 | 113,5911 | 0,9819 | 0,29730,0799 |
| ENSBTAG00000025258 | LOC515676 | 286,7710 | 1,1528 | 0,34940,0799 |
| ENSBTAG00000019975 | IL7R | 8,5639 | 1,1748 | 0,35610,0799 |
| ENSBTAG00000004034 | SESN3 | 201,9033 | 0,7658 | 0,23250,0800 |
| ENSBTAG00000000816 | PRDM1 | 1184,2791 | 0,9359 | 0,28430,0800 |
| ENSBTAG00000006355 | SLC30A2 | 11,3768 | 1,1879 | 0,36070,0800 |
| ENSBTAG00000017875 | ARHGAP30 | 56,9940 | 0,8100 | 0,24660,0809 |
| ENSBTAG00000034693 | SYT1 | 32,7892 | 1,1790 | 0,35880,0809 |
| ENSBTAG00000021919 | NAV1; LOC100849836 | 263,7395 | 0,4732 | 0,14450,0827 |
| ENSBTAG00000019472 | NR3C1 | 141,8328 | 0,5263 | 0,16060,0827 |
| ENSBTAG00000003014 | TRPV2 | 62,2923 | 0,8060 | 0,24610,0827 |
| ENSBTAG00000036078 | EMP1 | 267,2550 | 0,9118 | 0,27930,0848 |
| ENSBTAG00000011518 | NA | 44,6144 | 0,7337 | 0,22490,0854 |
| ENSBTAG00000017165 | MATN2 | 3288,7491 | 0,8039 | 0,24670,0858 |
| ENSBTAG00000017390 | LGI2 | 10,4923 | 1,1503 | 0,35410,0877 |
| ENSBTAG00000002469 | KANK3 | 381,5327 | 0,7453 | 0,23040,0910 |
| ENSBTAG00000015739 | MRC2 | 1581,8356 | 0,6636 | 0,20530,0911 |
| ENSBTAG00000007421 | CDH5 | 587,4520 | 0,7800 | 0,24160,0915 |
| ENSBTAG00000020638 | TIMP3 | 7435,1287 | 0,4903 | 0,15220,0926 |
| ENSBTAG00000021980 | RASSF8 | 397,3486 | 0,8203 | 0,25550,0949 |
| ENSBTAG00000005762 | LYNX1 | 50,4842 | 0,5947 | 0,18540,0955 |
| ENSBTAG00000010462 | Bt.110263 | 307,0571 | 0,6678 | 0,20880,0961 |
| ENSBTAG00000015032 | CD14 | 154,9510 | 0,7251 | 0,22680,0961 |
| ENSBTAG00000006789 | FGD5 | 488,5683 | 0,7416 | 0,23180,0961 |
| ENSBTAG00000006977 | PLP1 | 10,6436 | 1,1542 | 0,36070,0961 |
| ENSBTAG00000004797 | METTL19 | 119,9696 | 0,5944 | 0,18610,0967 |
| ENSBTAG00000020601 | ZNF366 | 66,4794 | 0,9198 | 0,28820,0970 |
| ENSBTAG00000011196 | C1QB | 130,3047 | 0,9398 | 0,29480,0978 |

**Supporting Table B.** List displaying all genes with up-regulated expression in uterine biopsies from pregnant cows (*Padj*<0.1). **id: Ensembl number identification; lfcSE: log2foldchange standard error; padj: p-value adjusted after Benjamini-Hochberg correction for multiple tests.**

| id | Gene.Symbol | baseMean | log2FoldChange | lfcSEpadj |
| --- | --- | --- | --- | --- |
| ENSBTAG00000012972 | CYP2U1 | 360,2653 | -0,5133 | 0,10250,0007 |
| ENSBTAG00000003423 | DDX24 | 1529,2548 | -0,2689 | 0,05930,0038 |
| ENSBTAG00000017502 | RIMKLA | 15,0968 | -1,3978 | 0,31330,0048 |
| ENSBTAG00000047970 | NA | 231,0191 | -0,4945 | 0,12100,0134 |
| ENSBTAG00000038361 | SERPINA11 | 40,2644 | -1,3242 | 0,32770,0142 |
| ENSBTAG00000020174 | HBS1L | 1200,4947 | -0,2902 | 0,07400,0192 |
| ENSBTAG00000004472 | DYNLT1 | 826,6046 | -0,4779 | 0,13270,0418 |
| ENSBTAG00000001246 | ATP1A1 | 17790,9222 | -0,5038 | 0,14020,0428 |
| ENSBTAG00000019291 | GRB14 | 350,9180 | -0,9473 | 0,26720,0487 |
| ENSBTAG00000012467 | MASP1 | 52,6749 | -1,2715 | 0,36070,0500 |
| ENSBTAG00000012909 | CRABP1 | 371,8436 | -0,7955 | 0,22790,0550 |
| ENSBTAG00000019696 | RCE1 | 467,4498 | -0,2908 | 0,08390,0568 |
| ENSBTAG00000004040 | UGT2A3 | 11,9588 | -1,1981 | 0,34680,0583 |
| ENSBTAG00000012059 | MVD | 731,9768 | -0,3596 | 0,10440,0595 |
| ENSBTAG00000011319 | SLTM | 1697,1665 | -0,2703 | 0,07850,0595 |
| ENSBTAG00000001485 | PPIP5K2 | 1187,9679 | -0,3459 | 0,10060,0600 |
| ENSBTAG00000006646 | PRMT1 | 1867,7276 | -0,2780 | 0,08140,0637 |
| ENSBTAG00000016465 | DHCR7 | 548,1538 | -0,5550 | 0,16270,0639 |
| ENSBTAG00000006759 | SUCLA2 | 1235,2989 | -0,2498 | 0,07360,0670 |
| ENSBTAG00000018973 | COPS3 | 1272,5499 | -0,2329 | 0,06920,0718 |
| ENSBTAG00000004266 | DDX25 | 12,0194 | -1,2070 | 0,36070,0742 |
| ENSBTAG00000006168 | RBM45 | 624,7814 | -0,2673 | 0,08000,0742 |
| ENSBTAG00000009818 | LOC615933 | 56,0469 | -0,7319 | 0,22020,0773 |
| ENSBTAG00000008755 | 41340,0000 | 1442,2441 | -0,3637 | 0,10950,0773 |
| ENSBTAG00000000177 | MSLN | 728,7746 | -1,0832 | 0,32980,0809 |
| ENSBTAG00000009518 | ALKBH3 | 166,9544 | -0,5452 | 0,16770,0877 |
| ENSBTAG00000014255 | CCDC132 | 637,3383 | -0,2933 | 0,09030,0877 |
| ENSBTAG00000032905 | LOC783226; RMDN2 | 267,7538 | -0,3734 | 0,11540,0910 |
| ENSBTAG00000038783 | NA | 12,0068 | -1,1283 | 0,34900,0910 |
| ENSBTAG00000009277 | RGS17 | 5,6467 | -1,1355 | 0,35220,0926 |
| ENSBTAG00000001527 | TMEM213 | 288,4157 | -1,1294 | 0,35050,0926 |
| ENSBTAG00000013591 | CYB5R4 | 247,6895 | -0,3286 | 0,10220,0941 |
| ENSBTAG00000007105 | PWP2 | 236,8216 | -0,3893 | 0,12120,0944 |
| ENSBTAG00000018372 | STARD7 | 1929,0116 | -0,2382 | 0,07440,0961 |
| ENSBTAG00000012718 | XK | 190,9380 | -0,7959 | 0,24890,0961 |
| ENSBTAG00000039446 | BTRAPPIN-4 | 649,2324 | -1,1394 | 0,35780,0983 |
