# Supplementary material for: The Transcriptome Signature of the Receptive Bovine Uterus Determined at Early Gestation
Source: PLoS One. 2015 Apr 7;10(4):e0122874. doi: 10.1371/journal.pone.0122874 (PMC4388694; doi:10.1371/journal.pone.0122874)
Supplement: S1 Materials — (DOCX) [file pone.0122874.s002.docx]

**S1 Materials.** Sequence Read Archive (SRA) accession numbers of the filtered reads resulted from the RNAseq. All reads sequences were deposited in the Sequence Read Archive (SRA) of the NCBI (http://www.ncbi.nlm.nih.gov/sra/; accession numbers in Supporting Table 1) and, an overview of the gene expression data has been deposited in NCBI’s Gene Expression Omnibus (GEO) and is accessible through GEO Series accession number GSE65117.

**Supplementary Table 1.** SRA accession numbers of the filtered reads resulted from the RNAseq.

| Accession | Sample NameLabel | Bioproject | Experiment number | Run number |
| --- | --- | --- | --- | --- |
| SAMN03246806 | P904Pregnant_endometrium904 | PRJNA268916 | SRX790889 | SRR1685974 |
| SAMN03246807 | P1541Pregnant_endometrium1541 | PRJNA268916 | SRX790896 | SRR1685980 |
| SAMN03246808 | P1631Pregnant_endometrium1631 | PRJNA268916 | SRX791942 | SRR1685980 |
| SAMN03246809 | P1753Pregnant_endometrium1753 | PRJNA268916 | SRX791948 | SRR1687092 |
| SAMN03246810 | P2396Pregnant_endometrium2396 | PRJNA268916 | SRX791950 | SRR1687093 |
| SAMN03246811 | NP2541Non-Pregnant_endometrium2541 | PRJNA268916 | SRX792027 | SRR1687099 |
| SAMN03246812 | NP238Non-Pregnant_endometrium238 | PRJNA268916 | SRX792034 | SRR1687175 |
| SAMN03246813 | NP617Non-Pregnant_endometrium617 | PRJNA268916 | SRX792036 | SRR1687176 |
| SAMN03246814 | NP638Non-Pregnant_endometrium638 | PRJNA268916 | SRX792037 | SRR1687179 |
| SAMN03246815 | NP856Non-Pregnant_endometrium856 | PRJNA268916 | SRX792039 | SRR1687182 |
| SAMN03246816 | NP2810Non-Pregnant_endometrium2810 | PRJNA268916 | SRX792043 | SRR1687192 |
